# Sheep and goats as indicator animals for the circulation of CCHFV in the environment

**DOI:** 10.1007/s10493-015-9996-y

**Published:** 2015-12-24

**Authors:** Isolde Schuster, Marc Mertens, Slavcho Mrenoshki, Christoph Staubach, Corinna Mertens, Franziska Brüning, Kerstin Wernike, Silke Hechinger, Kristaq Berxholi, Dine Mitrov, Martin H. Groschup

**Affiliations:** Institute of Novel and Emerging Infectious Diseases, Friedrich-Loeffler-Institut, Federal Research Institute for Animal Health, Greifswald, Isle of Riems Germany; Faculty of Veterinary Medicine, Saints Cyril and Methodius University in Skopje, Skopje, Macedonia; Institute of Epidemiology, Friedrich-Loeffler-Institut, Federal Research Institute for Animal Health, Greifswald, Isle of Riems Germany; Fachdienst Veterinärwesen und Verbraucherschutz, Landkreis Vorpommern-Rügen, Stralsund, Germany; Institute of Diagnostic Virology, Friedrich-Loeffler-Institut, Federal Research Institute for Animal Health, Greifswald, Isle of Riems Germany; Faculty of Veterinary Medicine in Tirana, Tirana, Albania

**Keywords:** Crimean-Congo hemorrhagic fever virus, Southeastern Europe, Ruminants, Prevalence rate, Risk area, CCHF

## Abstract

Crimean-Congo hemorrhagic fever virus (CCHFV) is a tick-borne virus, which causes a serious illness with case-fatality rates of up to 80 % in humans. CCHFV is endemic in many countries of Africa, Asia and Southeastern Europe. Next to the countries with endemic areas, the distribution of CCHFV is unknown in Southeastern Europe. As the antibody prevalence in animals is a good indicator for the presence or absence of the virus in a region, seroepidemiological studies can be used for the definition of risk areas for CCHFV. The aim of the present study was to reveal which ruminant species is best suited as indicator for the detection of a CCHFV circulation in an area. Therefore, the prevalence rates in sheep, goats and cattle in different regions of Albania and Former Yugoslav Republic of Macedonia were investigated. As there are no commercial tests available for the detection of CCHFV-specific antibodies in animals, two commercial tests for testing human sera were adapted for the investigation of sera from sheep and goats, and new in-house ELISAs were developed. The investigation of serum samples with these highly sensitive and specific assays (94–100 %) resulted in an overall prevalence rate of 23 % for Albania and of 49 % for Former Yugoslav Republic of Macedonia. Significant lower seroprevalence rates for CCHFV were found in cattle than in small ruminants in given areas. These results indicate that small ruminants are more suitable indicator animals for CCHFV infections and should therefore be tested preferentially, when risk areas are to be identified.

## Introduction

Crimean-Congo hemorrhagic fever virus (CCHFV) is a tick-borne virus, belonging to the genus *Nairovirus* of the family *Bunyaviridae.* CCHFV circulates in many countries of Africa, Asia and Southeastern Europe (Hoogstraal 1979). In Europe, its spread closely correlates with the distribution of *Hyalomma marginatum* ticks, which are both vectors and reservoirs of CCHFV (Whitehouse 2004). The northern distribution limit is the 46°N (Hubalek and Rudolf 2012). CCHFV circulates in an enzootic tick-vertebrate-tick cycle, but it can also be transmitted horizontally (co-feeding, venereal transmission, transstadial) and vertically (transovarial) within the tick population (Logan et al. 1989; Gonzalez et al. 1992).

*Hyalomma marginatum* ticks feed on various domestic (e.g. cattle, sheep, goats) and wild animals (e.g. hares, hedgehogs). Those species play an essential role in the amplification and spread of the virus as well as in the lifecycle of the ticks (Zeller et al. 1994). Although animals can develop a viremia lasting up to 2 weeks (Gunes et al. 2011), there is no evidence that a CCHFV infection results in any clinical sign in animals (Whitehouse 2004; Ergonul 2006). In contrast, CCHFV can cause a serious hemorrhagic disease in humans with case-fatality rates ranging from 5 % (Turkey; Yilmaz et al. 2008) to 80 % (China; Yen et al. 1985). Divergences in case-fatality rates among countries may be due to differences in circulating virus strains, the effectiveness of health care systems and in the education and awareness of the public (Maltezou et al. 2010). Statistical reasons may also play a role, as in Turkey even very mild cases of CCHFV are notified by the medical system and not only severe cases, which have a lower chance to survive in general.

CCHFV can be transmitted to humans by tick-bite and contact with blood, body fluids or tissues of viremic animals or humans. Nosocomial infections are frequently reported (Altaf et al. 1998; Mourya et al. 2012). No vaccination is available, and the treatment focusses mainly on supportive measures (Ergonul 2006). Within Southeastern Europe, human cases have been reported from Bulgaria, Republic of Kosovo, Albania and Greece (Papa et al. 2004, 2010, 2011; Avšič-Županc 2008). In addition, up to 1300 human cases have been reported from Turkey annually in the last decade (Maltezou et al. 2010). Next to those countries with endemic areas, nearly no information is available about the distribution of CCHFV in Southeastern Europe.

The status of CCHFV-specific antibodies in the animal population of a region is a good indicator for the presence or absence of CCHFV in the respective area (Hoogstraal 1979). However, there are no commercial assays available for the detection of CCHFV-specific antibodies in animals. Only a few in-house assays have been published, but in most cases information regarding the sensitivity and specificity of those assays is limited (Mertens et al. 2013).

In the present manuscript, the development of new in-house enzyme-linked immunosorbent assays (ELISAs) for the detection of CCHFV-specific antibodies in sheep and in goats is described. The aim was to develop low-cost screening tests, which can be established at partner laboratories in multiple countries. Further, a commercial ELISA and a commercial immunofluorescence assay (IFA), which have been developed for the examination of sera from humans, were adapted for use as confirmation assays. Using these assays, serum samples from sheep and goats from five regions of Albania and three regions of Former Yugoslav Republic of Macedonia were investigated. Samples from cattle, previously published assays were used (Mertens et al. 2015). Finally, the prevalence rates in cattle, sheep and goats were compared to identify which ruminant species is best suited as indicator for the detection of a CCHFV circulation in an area.

## Materials and methods

### Serum samples from Albania and from the Former Yugoslav Republic of Macedonia

For the seroepidemiological survey, 534 serum samples from cattle, sheep and goats were collected in Albania in the districts of Berat, Has, Kolonjë, Lezhë and Pogradec in 2011 and 2013. The serum samples from cattle from the districts of Kolonjë, Lezhë and Pogradec were part of previous publications (Lugaj et al. Lugaj et al. 2014a, b, c). Furthermore, 330 serum samples from cattle, sheep and goats were collected in the Former Yugoslav Republic of Macedonia in the Northeast region, the Vardar region and the Southeast region between 2009 and 2011. The serum samples from cattle were part of a study which was published previously (Mertens et al. 2015).

### Adaptation of a commercial CCHFV ELISA and IFA

A commercial ELISA for the detection of CCHFV-specific antibodies in human sera (Vector Best, Novosibirsk, Russia) was adapted for testing sera from sheep and goats. All washing steps were performed with PBS-Tween20. Sera were diluted 1:100 in dilution buffer (90 % SDB- and 10 % SPSD-buffer of the manufacturer). Diluted sera (100 μl/well) were incubated for 1 h at 37 °C. After washing the plates, 100 μl/well of rabbit-anti-sheep-IgG-HRP conjugate (Southern Biotech, Birmingham, AL, USA) or rabbit-anti-goat-IgG-HRP conjugate (Southern Biotech) were added. Both were diluted 1:6000 in conjugate dilution buffer (by manufacturer) and incubated for 30 min at 37 °C. After washing the plates, 100 μl tetramethylbenzidine (TMB, Bio-Rad, Munich, Germany) solution was added per well, and the reaction was stopped with H_2_SO_4_ after 10 min. The extinction was measured at λ = 450 nm (reference wavelength 620 nm). Serum samples with OD-values (background reaction subtracted) of 0.5 and lower were defined as ‘negative’, whereas serum samples with OD-values of 0.6 and 0.7 were defined as ‘inconclusive’ and serum samples with OD-values of 0.8 and higher were defined as ‘positive’.

A commercial IFA (Euroimmun, Lübeck, Germany) for the detection of CCHFV-specific antibodies in human sera was adapted to sheep and goats. The assay is based on transfected cells expressing the CCHFV glycoprotein c (Gc) and cells expressing the nucleocapsid protein (N-protein). All incubation steps were performed for 30 min at 37 °C, and slides were washed for 5 min in PBS containing 0.1 % Tween20. Aliquots of 25 µl of sheep and goat serum dilutions [1:20 in TBST buffer (0.05 M Tris, 0.138 M NaCl, 0.0027 M KCl, 0.1 % Tween20; pH 10)] were applied to each well on the slide, respectively. After washing, 20 μl of rabbit-anti-sheep-IgG-FITC conjugate (Southern Biotech) diluted 1:320 or 20 µl of donkey-anti-goat-IgG-FITC conjugate (Genetex, Hsinchu City, Taiwan) diluted 1:200 in TBST (containing 0.005 % Evans Blue) were added to each well. Following another washing step, glycerin was added and the results were visualized with a fluorescence microscope. The commercial ELISA and IFA protocols for testing sera from cattle have been published previously (Mertens et al. 2015).

### In-house CCHFV ELISAs for the detection of CCHFV-specific antibodies

Sera from cattle were screened using a previously published protocol, which is based on His-tagged recombinant N-protein of CCHFV-strain Kosovo Hoti (Accession no. DQ133507) as antigen (Mertens et al. 2015). This protocol was slightly modified for testing sheep and goat sera. One half of the wells of 96-Well MaxiSorp immunoplates (Nunc, Roskilde, Denmark) were coated with 100 µl/well coating buffer (PBS, 1 % BSA; pH 8.0) containing 0.2 μg antigen and the second half without antigen for 1 h at 37 °C. For testing sera from sheep, 200 µl/well blocking buffer (PBS, 0.05 % Tween 20, 3 % BSA; pH 8.0) were added and the plates were incubated for 1 h at 37 °C. The plates were washed 4 × with 250 µl/well PBS-Tween 0.1 %. Sheep sera were diluted 1:40 in serum dilution buffer (IDVet, Grabels, France) and 100 μl/well was added in duplicate to wells coated with and without antigen and incubated for 1 h at 37 °C. Before being diluted, the serum samples were centrifuged for 5 min at 8000*g*. After washing, 100 µl/well of Protein G-conjugate (Calbiochem, San Diego, CA, USA), diluted 1:500 in conjugate dilution buffer (IDVet), were added, and the plates were incubated for 2 h at 37 °C. All incubation steps were carried out in an incubator containing 5 % CO_2_. After a washing step, 100 μl/well TMB solution (Bio-Rad) was added and the reaction was stopped with H_2_SO_4_ after 30 min. The absorbance was measured at λ = 450 nm (reference wavelength 620 nm). When goat sera were tested, a different blocking solution (IDVet) and an anti-ruminant-conjugate (IDVet), diluted 1:10 in conjugate dilution buffer (IDVet), were used.

The result (R) for each serum sample was calculated by subtracting the mean value of the OD-values of the two wells without antigen from the mean value of the OD-values of the two wells coated with antigen. The final result (fR) for each sample was calculated by dividing the final OD-value of a sample by the final OD-value of the positive control sample (fR = [R-sample/R-positive] * 100).

Serum samples from sheep with fR < 15 % were classified as ‘negative’, with fR > 28 % as ‘positive’ and with fR between 15 and 28 % as ‘inconclusive’. Serum samples from goats with fR < 12 % were classified as ‘negative’, with fR > 22 % as ‘positive’ and with fR between 12 and 22 % as ‘inconclusive’.

### Validation of the assays

Since no gold-standard assay exists in the serological diagnostic of CCHFV, the adapted commercial assays were validated by comparative analysis. The adapted commercial IFAs, as imaging procedures, were used as reference tests for the validation of the adapted commercial ELISAs. For the validation of the IFAs themselves, the adapted commercial ELISAs as well as the in-house ELISAs were used as reference tests. Only serum samples which gave a positive signal in both ELISAs, were defined as ‘positive’ and were included in the validation of the IFAs. Both, the adapted commercial and the in-house ELISA are based on completely different antigens. Therefore, a matching result was considered to be valid. For the validation of the in-house ELISAs, the adapted commercial IFAs and ELISAs were used as reference assays for the characterization of a positive serum panel. Because there is no evidence for the circulation of CCHFV in Germany, serum samples from this country were used as negative control samples.

For the validation of the adapted commercial ELISAs, 81 positive sheep serum samples from Albania and from Former Yugoslav Republic of Macedonia and 73 positive goat serum samples from Former Yugoslav Republic of Macedonia were used as positive reference sera. Another 138 sheep and 197 goat sera from Germany were included as negative reference samples. For the validation of the adapted commercial IFAs, 78 positive serum samples from sheep and 72 positive serum samples from goats were used. In addition, 23 negative serum samples from sheep and 32 negative serum samples from goats from Germany were included. For the validation of the in-house CCHFV-sheep ELISA, 78 positive serum samples from sheep from Albania and Former Yugoslav Republic of Macedonia, as well as 138 negative serum samples from sheep from Germany were used. The in-house CCHFV-goat ELISA was validated using 50 positive sera from the Former Yugoslav Republic of Macedonia and 197 negative sera from Germany.

### Seroepidemiological studies

For the seroepidemiological studies a flow-chart was used to classify the sera from cattle, sheep and goats from Albania and from Former Yugoslav Republic of Macedonia as ‘positive’, ‘negative’ and ‘inconclusive’ (Mertens et al. 2009). Briefly, samples tested ‘positive’ or ‘inconclusive’ in the in-house ELISAs were retested in the adapted commercial ELISAs. Samples giving discrepant results in these two assays were tested in the adapted commercial IFAs for a final conclusion.

### Statistical analysis

The comparison of prevalence rates was based on Fisher’s Exact Test. The data were corrected using the Bonferroni method (Noordhuizen et al. 2001). The tests were done on the significance level of *p* < 0.05 (95 % confidence interval). The Bonferroni correction (*p* value/no. of statistical tests), lowered the test significance level to 0.01.

## Results

### Validation of the assays

The validation of the adapted commercial ELISAs resulted in diagnostic sensitivities of 98 and 100 % and diagnostic specificities of 100 % for sheep and goats (Table 1). For sera from sheep, the validation of the adapted commercial IFA resulted in a diagnostic sensitivity of 99 % and diagnostic specificity of 100 %. For sera from goats, the diagnostic sensitivity was 100 % and the diagnostic specificity was 94 % (Table 1); 3 % of the sheep sera and 5 % of the goat sera gave inconclusive results in the IFAs.

**Table 1:** Validation of the commercial and in-house enzyme-linked immunosorbent assays (ELISA) and immunofluorescence assays (IFA)

	Diagnostic sensitivity (%)	Diagnostic specificity (%)
*Commercial ELISA*		
Sheep	98 (91–100)	100 (97–100)
Goat	100 (95–100)	100 (98–100)
*Commercial IFA*		
Sheep	99 (93–100)	100 (84–100)
Goat	100 (95–100)	94 (79–99)
*In*-*house ELISA*		
Sheep	96 (89–99)	98 (93–100)
Goat	98 (88–100)	96 (92–98)

95 % confidence intervals are shown in parentheses

The diagnostic sensitivity of the in-house CCHFV-sheep ELISA was 96 % and the diagnostic specificity 98 % (Table 1), with 5 % of the sera giving ‘inconclusive’ results (Table 2). The validation of the in-house CCHFV-goat ELISA resulted in a diagnostic sensitivity of 98 % and diagnostic specificity of 96 % (Table 1); 5 % of the serum samples gave inconclusive results (Table 2).

**Table 2:** Results of the in-house enzyme-linked immunosorbent assays (ELISA) for the reference sera

	Positive reference sera	Negative reference sera
*Sheep*		
Positive	74	3
Negative	3	126
Inconclusive	1	9
*Goats*		
Positive	45	8
Negative	1	180
Inconclusive	4	9

### Seroepidemiological study

The overall CCHFV antibody seroprevalence rate for ruminants from Albania was 23 %. It ranged from 0 to 10 % in the cattle population, from 0 to 97 % in the sheep population and from 0 to 89 % in the goat population (Table 3). In the Former Yugoslav Republic of Macedonia, an overall seroprevalence rate of 49 % was found. The prevalence rates in the cattle population ranged from 0 to 80 %, in the sheep population from 44 to 86 % and in the goat population from 0 to 75 % (Table 3).

**Table 3:** Seroepidemiological studies in Albania and the Former Yugoslav Republic of Macedonia

Location of collection	Antibody detection								
	Region	Total		Cattle		Sheep		Goats	
		n	Prevalence (%)	n	Prevalence (%)	n	Prevalence (%)	n	Prevalence (%)
Albania	Berat	74	16 (9–27)	31	0 (0–15)	18	67 (41–87)	25	0 (0–17)
	Has	183	20 (15–27)	108	10 (5–18)	51	25 (14–40)	24	54 (33–74)
	Kolonjë	92	43 (33–54)	54	7 (2–18)	29	97 (82–100)	9	89 (51–100)
	Lezhë	157	18 (13–25)	42	5 (1–16)	37	35 (20–53)	78	18 (10–28)
	Pogradec	28	25 (11–45)	6	0 (0–53)	7	0 (0–48)	15	47 (21–73)
	Total	534	23 (20–27)	241	7 (4–11)	142	46 (38–55)	151	28 (21–36)
Former Yugoslav Republic of Macedonia	Northeast	92	57 (46–67)	20	80 (56–94)	25	44 (24–65)	47	53 (38–68)
	Vardar	76	61 (49–72)	23	17 (5–39)	49	86 (73–94)	4	0 (0–66)
	Southeast	162	40 (32–47)	80	0 (0–6)	50	80 (66–90)	32	75 (57–89)
	Total	330	49 (44–55)	123	16 (10–24)	124	75 (66–82)	83	59 (48–70)

n: number of samples; 95 % confidence intervals are shown in parentheses

## Discussion

Here, we report the adaptation and validation of diagnostic assays for the detection of CCHFV-specific IgG-antibodies in sheep and in goats and their use in seroepidemiological studies in Albania and Former Yugoslav Republic of Macedonia. High CCHFV seroprevalence rates were found for most of the examined regions, with average rates of 23 % for Albania and 49 % for Former Yugoslav Republic of Macedonia. The high prevalence rates of CCHFV-specific antibodies in ruminants in the two countries indicate a high risk for human infections in various regions. In Albania, the endemic regions Kukes and Has are well known and characterized (Papa et al. 2009). The high prevalence rates in the ruminant populations of other areas (Berat, Kolonjë, Lezhë, Pogradec) and the occurrence of sporadic human cases in several regions might be an indication that CCHFV circulates in the whole country (Papa et al. 2002). For the Former Yugoslav Republic of Macedonia no human cases were reported to date. A retrospective study revealed the presence of CCHFV in ticks, and in a previous study the high CCHFV-specific antibody prevalence in cattle was discussed (Vesenjak-Hirjan et al. 1991; Mertens et al. 2015). However, further investigations are necessary to fully assess the risk of human infections (Mertens et al. 2013).

Prevalence rates in small ruminants and cattle were quite distinct (*p* < 0.01) within the studies. This was most obvious for sera from the region of Kolonjë (Albania), where 97 % of the sheep and 89 % of the goats tested positive for CCHFV-specific antibodies, but only 7 % of the cattle (*p* < 0.01). Likewise, high prevalence rates in sheep (80 %) and in goats (75 %) were found in the Southeastern region of Former Republic of Macedonia, where none of the cattle sera gave a positive reaction (*p* < 0.01). These findings are in line with results of previous studies from Turkey, Egypt, Iraq, Iran, Oman and Saudi Arabia, in which higher antibody prevalence rates have been found in small ruminants than in cattle (Tantawi et al. 1981; Williams et al. 2000; Mohamed et al. 2008; Telmadarraiy et al. 2010; Tuncer et al. 2014).

In some cases we found also big differences between the prevalence in sheep vs. goats. In Berat (Albania), the prevalence in sheep was very high (67 %), but no goat was positive for CCHFV-specific antibodies (*p* < 0.01). In general, the prevalence rate in Albania was significantly higher in the sheep than in the goat population (46 vs. 28 %; *p* < 0.01). As this finding was not confirmed in the Former Yugoslav Republic of Macedonia (*p* > 0.01), it may be an artifact caused by sampling and/or the number of collected samples. However, despite of these potential deficits, the accumulated data indicate a clear trend of higher seroprevalence rates in small ruminants than in cattle.

The results of our study highlight the suitability of small ruminants as indicator animals for seroepidemiological CCHFV monitoring studies to determine the presence or absence of CCHFV in a given region. This is surprising as observations support the idea that cattle are the preferential feeding host for adult *H. marginatum* ticks (Hoch et al. 2015). The knowledge about the mechanisms governing the dynamics of CCHFV circulation in a suitable habitat and the role of the various animals is very limited. Some studies have demonstrated an accumulation of the antibody prevalence by increasing age of the tested animal population (Wilson et al. 1990; Barthel et al. 2014). The life span of the animal species, husbandry conditions, usage of repellents, host-preferences of the ticks and susceptibility of animal species and breeds for CCHFV infections may also play a role. Anyhow, further investigations of the situation in other countries, on animal infections and on tick-host interactions are necessary and may help to improve our understanding of the transmission cycle of CCHFV.
